# The Peterborough Exemplar: a protocol to evaluate the impact and implementation of a new patient-centred, system-wide community mental healthcare model in England

**DOI:** 10.1186/s12961-022-00819-0

**Published:** 2022-02-05

**Authors:** Lida Efstathopoulou, Grace Jagger, Jules Mackenzie, Kathryn Faulkner, Trish Barker-Barrett, Rory Cameron, Adam P. Wagner, Jesus Perez

**Affiliations:** 1 NIHR Applied Research Collaboration–East of England (ARC EoE), Douglas House, 18 Trumpington Road, Cambridge, CB2 8AH United Kingdom; 2 Cambridgeshire and Peterborough NHS Foundation Trust, Elizabeth House, Fulbourn, Cambridge, CB21 5EF United Kingdom; 3 Cambridgeshire County Council and Peterborough City Council, Scott House, 5 George Street, Huntingdon, PE29 3AD United Kingdom; 4 Norwich Medical School, University of East Anglia, Norwich, NR4 7TJ United Kingdom; 5 Department of Psychiatry, University of Cambridge, Herchel Smith Building, Robinson Way, Cambridge, CB2 0SZ United Kingdom

**Keywords:** Community mental healthcare model, Adult mental health services, Service evaluation

## Abstract

**Background:**

Community mental healthcare has significantly grown since de-institutionalization. Despite progress, service fragmentation and gaps in service provision remain key barriers to effective community care in England. Recent mental healthcare policies highlighted the need to transform service provision by developing patient-centred, joined-up community mental healthcare. In response to policy guidance, a system-wide community mental healthcare model was developed in Peterborough (England). The “Peterborough Exemplar” is based on two main pillars: (1) the creation of knowledge exchange pathways to strengthen interorganizational relationships, and (2) the development of new, accessible community services addressing existing service gaps. This paper presents the protocol developed to evaluate the Peterborough Exemplar.

**Methods:**

A quasi-experimental design with an intervention group and a nonequivalent comparator group has been developed to compare service provision provided in Peterborough pre- and post-intervention with services provided in Fenland, a neighbouring area where service users access usual care. Two evaluation methods will be employed to compare service provision between the two groups: (1) outcome measures completed by service users and carers will be analysed to assess quality of life and service satisfaction, and (2) service activity data will be analysed to assess service usage. In addition, qualitative interviews will be conducted with staff members of participating organizations to explore the implementation of the Exemplar in Peterborough and evaluate knowledge exchange processes among local service providers. A matched control approach will be used to compare outcome measures between the two areas. Descriptive and inferential statistics, including chi-square tests, will be used to analyse service activity data and examine differences between the two areas. A thematic analysis will be adopted to analyse qualitative data.

**Discussion:**

Outcomes of the evaluation will contribute to understanding the contribution of the Peterborough Exemplar on mental health service provision locally. Evaluation findings and intermediate reporting will be shared with organizations involved in the implementation of the Peterborough Exemplar and with local decision-makers to inform the Exemplar delivery. As the Peterborough Exemplar is an Early Implementer (EI) site funded by NHS England, findings will be shared with policy-makers to inform national policy on community mental healthcare and integrated care.

**Supplementary Information:**

The online version contains supplementary material available at 10.1186/s12961-022-00819-0.

## Background

Following de-institutionalization, community mental healthcare gradually became the main form of mental health service provision, combined with hospital care, where needed [1]. Community mental healthcare is based on provision of care within the community, where individuals’ mental health and well-being can be supported by various professionals and service providers [1]. An effective community mental healthcare approach takes a system-wide approach and considers the characteristics of local populations, together with individual rights, experiences and needs, when planning care [1, 2]. Evidence shows that community mental healthcare systems differ across Europe, as progress on developing effective care systems depends on the development of national mental health policies, funding allocated to mental healthcare, local mental health needs, the willingness of national and local decision-makers to implement community mental healthcare, and the availability of sufficient evidence on the impact of community mental health services [2, 3].

In England, transition from institutionalized care to community mental healthcare was marked by the creation of community mental health teams (CMHTs) [4]. CMHTs were established to offer access to coordinated care that is appropriate to service users’ needs and is provided by staff with diverse professional backgrounds [4]. Community mental healthcare gradually became an umbrella term encompassing services ranging from early-intervention psychological therapies to treatment of complex needs and crisis support. Examples of specialist community services include crisis resolution teams (CRTs), Improving Access to Psychological Therapies (IAPT) services, assertive community treatment teams (ACTs) and liaison psychiatry services [5–8]. New community services were also developed to offer treatment for specific mental health needs, namely early intervention in psychosis (EIP), eating disorders (ED) and personality disorders (PD) [9–11]. As a result, a range of community mental health services have been developed and continue to emerge, with the aim of responding to the needs of local populations.

The collaboration between the National Health Service (NHS), social care and the third (voluntary) sector has long been recognized as a key component in the delivery of system-wide mental healthcare. Existing evidence shows that the development of partnerships between mental and social care can contribute to providing holistic care. as they can enable interprofessional collaboration, exchange of knowledge and information, more targeted investment of resources and a less complicated system of services [12]. The third sector has had a substantial impact on providing services that contribute to individual mental health and well-being, ranging from mental health support to housing services and service user advocacy, and has gradually received increasing public funds to deliver them [13]. Consequently, social care and third-sector organizations have been increasingly involved in the delivery of public mental healthcare provision.

Despite progress, community mental healthcare continues to face challenges. While the creation of additional specialist mental health services addressed a wide range of mental health needs, it also led to the development of a complicated service system, where services are often disconnected from each other [14]. To date, service users continue to experience a fragmented system of care, where waiting times to starting treatment can vary significantly; for instance, in 2018/2019 service users waited from 4 to 62 days to access IAPT services [15]. Parity of esteem between mental and physical healthcare is yet to be achieved, leaving community mental healthcare with comparatively limited financial resources to support local demand [16]. It is of note that the aforementioned challenges are thought to have been further exacerbated in the face of the pandemic’s impact on mental health and service provision [17]. Countries across the world had to rapidly adjust mental health policies to respond to mental health demand increased by the pandemic outbreak [17].

To address challenges in the quality of mental health service provision, the 2019 NHS Long Term Plan proposed to “redesign and reorganise core community mental health teams to move towards a new place-based, multidisciplinary service across health and social care aligned with primary care networks” [18, p. 69]. The subsequent Community Mental Health Framework for Adults and Older Adults (2019) expanded on this recommendation and called for the development of system-wide community mental healthcare based on the needs of local populations [19]. In response to those policies and the targets set by the local sustainability and transformation plan (STP), local service providers and funders in Cambridgeshire and Peterborough (England) developed and implemented the “Peterborough Exemplar” with the aim of strengthening local community mental healthcare [20]. The Exemplar received funding from NHS England as an early implementer (EI) of a new community care model [19].

## The Peterborough Exemplar community mental healthcare model

The components of the Peterborough Exemplar were developed based on the needs of the local population. Local intelligence was used by the Exemplar partners to identify parts of the service system that are most challenged. Information sources used included (i) data on local deprivation, health and mental health prevalence and service utilization; (ii) consultation with key stakeholder groups across the system, including general practitioners (primary care), mental health professionals (secondary care), local third-sector organizations and social care; and (iii) service user and carer feedback. Two main areas of intervention were identified as an outcome of this exercise, which formed the main pillars of the Exemplar: (1) interorganizational relationships and (2) service development based on local demand.

The first pillar of the Peterborough Exemplar aimed to systemize knowledge exchange and improve relationships between local service providers. Specifically, the pillar introduced the following: routine clinical meetings between primary and secondary care, called “virtual clinics”; a liaison team offering advice and support across the system for community-based interventions; mental health professionals providing expertise to the primary care mental health service (PCMHS) for assessing patients with complex needs; and a website strengthening information dissemination by collating local mental health and well-being resources. The second pillar focused on developing new services with lower access thresholds, including services on psychological skills, PDs and peer support, services for individuals with a dual diagnosis, a team supporting engagement with social prescribing, a mental health pharmacist and a social care pathway operating at a primary care level. The peer support groups, dual diagnosis service, engagement with social prescribing and social care pathway are jointly organized and delivered by the mental health trust, third-sector organizations and social care, demonstrating the implementation of elements of integrated care. Additional file 1: Table S1 presents a description of each of the pillars and components of the Peterborough Exemplar.

Programme theory was used during the development of the Exemplar to demonstrate the intervention’s impact on the local service system. It helped to explain links between the intervention components and the expected short- and long-term outcomes of this complex intervention [21, 22].

## Design

The composition of the Peterborough Exemplar was based on the following: (1) the creation of new services (e.g. new services for PDs and psychological needs), (2) the establishment of interorganizational processes (e.g. multi-agency meetings, liaison role of professionals) and (3) the involvement of multiple service providers. It can thus be defined as a complex intervention consisting of “interactive components” at intra- and interorganizational levels aimed at improving organizational performance and patient outcomes [23, p. 1, 24, 25].

Four main objectives were identified to assess the impact of the Peterborough Exemplar on mental health service provision and its implementation:

(i) Evaluating service user and carer outcomes on quality of life and service satisfaction
(ii) Examining service activity across the local health services system
(iii) Assessing the economic impact of the Exemplar
(iv) Exploring the implementation process of the Exemplar.

To address the evaluation objectives, a quasi-experimental design comparing service provision in two geographical areas and a qualitative method exploring Exemplar implementation have been employed. Specifically, the evaluation will adopt a quasi-experimental design by comparing an intervention group with a nonequivalent comparator group pre- and post-intervention [27]. Evaluations of complex interventions can follow both experimental and non-experimental designs depending on the structure of the intervention and the context within which it is implemented [23, 26]. Adoption of non-experimental designs is recommended when the implementation of an intervention is determined by policies, service demand or patient needs, rather than by researchers or when the implementation precedes the development of an evaluation [23, 26]. Quasi-experimental designs are observational designs appropriate for assessing “real-world effectiveness” and where health service use is based on clinical decision and patient choice, rather than on researcher’s decision via randomization [27, p. 39]. Using qualitative methods can provide valuable insights into the processes of implementation and can contribute to identifying elements about a complex intervention that may not have been initially considered [24].

This study was approved by and registered with the official NHS Quality Improvement Programmes of the Cambridgeshire and Peterborough NHS Foundation Trust and confirmed as service evaluation by the Health Research Authority of the United Kingdom [28]. Participants in qualitative interviews will receive a participant information sheet and a consent form to complete if they wish to participate in the evaluation. Data analysis follows the guidelines established by the United Kingdom Anonymisation Standard for Publishing Health and Social Care Data [29].

## Study setting

### Description of geographical areas

The study will compare service provision in two geographical areas. Services provided in Peterborough where the Exemplar is implemented are defined as the intervention group and will be compared with local services in Fenland, which was selected as the nonequivalent comparator group. Fenland is a neighbouring area where service users access usual care. Peterborough and Fenland areas are part of the Cambridgeshire and Peterborough County located in the East of England (United Kingdom). The nonequivalent group (Fenland) was selected among the six areas of the county as the most suitable area to be compared with Peterborough based on similarities in the populations between the two areas and service provision, allowing assessment of whether the intervention impacted the area where it was implemented [30].

Out of the 62 most relatively deprived lower-layer super output areas (LSOAs) (i.e. small population area) existing in Cambridgeshire and Peterborough County, 46 LSOAs are in Peterborough, and 11 LSOAs are in Fenland [31]. A multimorbidity analysis conducted by local authorities showed that both Peterborough and Fenland present a statistically significantly higher rate of physical and mental health comorbidity (8.5.% and 10.0%, respectively) compared to the county’s average (7.5%), and a statistically significantly higher rate in the prevalence of two or more morbidities in adults (25–84 years old) in comparison with the county’s average [32]. The two areas, together with the area of Huntingdon (located in the west of the county), present statistically significantly higher prevalence rates for health conditions including stroke and hypertension, coronary and heart disease, along with higher rates for dementia, depression and learning disabilities compared to the county’s average [33]. The total population of the Peterborough area is 184 000 residents, and Fenland has 95 000 residents, as recorded in the 2011 Census [34, 35]. Population differences will be taken into consideration in the analysis stage. Fenland is a rural area, while Peterborough is partially rural; thus, differences in geographical characteristics may affect access to services. However, service provision has also been offered online since the beginning of the COVID-19 pandemic, and online offers will likely continue to be available.

### Local mental health service provision

Mental health services across the county are provided by one NHS mental health trust (the Cambridgeshire and Peterborough NHS Foundation Trust). Social care and third-sector organizations also offer services that support mental health. Third-sector organizations may be local charities or local branches of national or regional charities. Primary care is provided by general practices (GPs) operating in each geographical area. Primary care has recently been organized in coalitions, called primary care networks (PCNs), that enable coordination of care.

To evaluate the impact of the Peterborough Exemplar across the local area, multiple levels of care were considered. The study design includes services introduced or redesigned by the Peterborough Exemplar, such as the PCMHS and the psychological skills services (PSS), as well as existing services that may be impacted as an outcome of the Exemplar implementation, for example the Trust’s mental health adult specialist services. Fenland services included in the study design were selected by identifying corresponding services between the two areas. Table 1 presents the services that will be included in the evaluation.

**Table 1:** Services included in the evaluation of the Peterborough Exemplar

Level of care	Service	Intervention (Peterborough)	Nonequivalent comparator (Fenland)
Primary care—physical health	General practitioners	✓	✓
Primary care—mental health	Primary care mental health service	✓	✓
Primary care—mental health	Psychological skills services Personality disorders community service Dual diagnosis service Social care pathway	✓	–
Secondary care—mental health	Adult specialist services providing mental healthcare	✓	✓
Social care	Social care pathway for specialized needs	✓	✓
Third sector	Third-sector organizations	✓	✓

## Involvement of stakeholder groups

Feedback from stakeholder groups informed the evaluation design. Two patient and public involvement (PPI) groups were organised with service users to discuss “What questions should we be asking to best evaluate if the services work for you?” Participants made suggestions on the parts of the system that were important to evaluate, highlighting communication between primary and secondary care, recovery pathways and service satisfaction. A further PPI group will be held to inform service users on how their feedback was used and to discuss further recommendations. In addition, individual meetings with frontline staff and managers were held to discuss the structure of the services and receive recommendations on the evaluation objectives. Feedback from staff members was also used to identify suitable quantitative measures and informed the guide for qualitative interviews. Finally, recommendations from the Exemplar steering group were used to inform the objectives of the evaluation.

## Methods

Three methods will be employed to assess the impact of the Peterborough Exemplar on service provision as well as the implementation process of the intervention. Specifically, two methods will be adopted as part of a quasi-experimental design comparing service provision in Peterborough and in Fenland. Routine outcome measures (1) will be analysed to assess quality of life and service experience of service users and carers. Routine service activity data (2) will be analysed to evaluate service utilization. Analysis of outcome data and service activity will be based on a comparison between the intervention and comparator groups pre- and post-intervention, namely a period of 18 months (October 2020–March 2022). Data will be collected in four time periods: *T*0 (October 2020–baseline), *T*1 (April 2021), *T*2 (October 2021) and *T*3 (April 2022). In addition to the final analysis, data will be analysed at the end of each period to assess the impact of the Exemplar and inform its delivery (see Additional file 2).

Qualitative semi-structured interviews (3) will be conducted to explore the implementation process of the Exemplar in Peterborough. Collection and analysis of qualitative data will be conducted throughout the 18-month evaluation period (see Additional file 2). Following the analysis of qualitative and quantitative data, a data triangulation plan will be generated, allowing assessment of the intervention using multiple data sources [36]. The selection of evaluation methods was also informed by exemplar programming theory, which outlined the target outcomes of the Peterborough Exemplar. The next sections detail each evaluation method.

1. Evaluation of service user and carer outcomes
  Routinely collected outcomes measures completed by service users accessing the Exemplar services and the secondary care specialist teams will be analysed pre- and post-treatment. Quality of life and care satisfaction of service users will be assessed using the DIALOG tool, an 11-item measure combining eight questions on satisfaction with quality of life and three on satisfaction with service provision [37]. A brief questionnaire with four further questions on service satisfaction and the impact of the COVID-19 pandemic is also included, as the Exemplar implementation began during the pandemic period. The EuroQol EQ-5D-5L will also measure quality of life, for use in the economic analysis of the Exemplar [38]. Finally, a brief carer-rated service satisfaction measure will be included in the evaluation to assess carers’ satisfaction with services. The carer measure has been developed within the Trust in collaboration with carers and is used to routinely evaluate carer satisfaction with provided care.
  Local intelligence used by the Exemplar partners to identify investment priorities highlighted service demand for PDs in particular. To assess the impact of the personality disorders community service (PDCS), the Standardized Assessment of Severity of Personality Disorder (SASPD) measure [39] will be included in the evaluation. The SASPD is completed by service users at the PDCS pre-treatment and post-treatment in both the intervention and comparator groups. The SASPD measure will be used to assess the severity level of the PDs of service users accessing the new Exemplar PD services (intervention group) and compared with the severity level of those accessing PD services in Fenland (comparator group).
  Measures included in the evaluation are part of clinical routine practice in both the intervention and comparator groups. A matched control approach will be adopted to compare outcomes measures between the intervention and comparator groups. In this approach, study participants between the two groups are matched based on key covariates by using, for instance, nearest-neighbour matching or weighting techniques [40]. When randomization is not possible (e.g. in a quasi-experimental design), a matched control approach allows for control of external factors that can affect the relation between an intervention and its outcome and strengthens internal validity [40, 50]. Matching between intervention and comparator groups will be based on independent variables, including age, gender, employment, ethnicity and service provided.
  Descriptive statistics will also be used to describe baseline characteristics of service users in the two areas. Missing responses in outcomes measures will be managed based on analytical frameworks of the selected measures (i.e. DIALOG, EQ-5D-5L and SASPD). A detailed statistical analysis plan is currently being developed to demonstrate the rationale and steps of the matched control analysis.
2. Evaluation of service activity
  Service activity will be analysed to assess service usage and examine differences between the intervention and comparator groups pre- and post-intervention. Specifically, routinely collected service activity data of service users who had at least one contact in a team within the Adult Specialist Directorate of the Mental Health Trust will be analysed for both geographical areas over the 18-month evaluation period. Service activity data will include demographic variables (e.g. age, gender) and service activity variables (e.g. referral source, dates of appointments, treatment team and discharge reason). Service activity data from other geographical areas or from other Trust directorates will be excluded. Aggregated data on service activity will also be obtained from Exemplar partners (social care, third-sector and primary care services). Those will include the following: (i) primary care—the number of patients seen by GPs and percentage of patients visiting the GP with a mental health need; (ii) social care—the number of new contacts and percentage of new enquiries for people with primary support reason mental health; and (iii) third sector—attendance in the peer support groups for PDs. Aggregated data will increase our understanding of service demand in the intervention and comparator groups. All quantitative data will be saved in electronic folders of the password-protected virtual work environments of the lead author and the economist researcher.
  Descriptive statistics will be used to present the characteristics of the patient cohort contained in service activity datasets, including percentages of demographic characteristics, services accessed by patients and diagnosis and to assess completeness of data. Changes in trends in key service utilization indicators during the 18-month evaluation period (*T*0–*T*4) between the two study groups will be analysed using *χ*^2^ (chi-square) tests. Service activity indicators include referral sources, number of contacts and access to specialist services in secondary care. Stata software (version 16) will be used to conduct the statistical analysis [41]. A detailed statistics and health economic analysis plan is currently being developed that outlines the statistical analysis of service usage.
  Taking into consideration data collection and analysis requirements of the selected methods and the resources available to the evaluation, assessing the Exemplar’s impact on service users focused on quantitative methods only (routine outcome measures and service activity usage). Quantitative methods allow data collection from a larger cohort of service users, are routinely collected within the Mental Health Trust and are less time-consuming for service users. However, qualitative methods, such as semi-structured interviews, can offer in-depth understanding of service users’ experience with service provision and complement quantitative findings in assessing the impact of complex interventions [24]. Collection of qualitative data from service users will be explored if the evaluation funding is extended.
3. Evaluation of the implementation process
  Qualitative interviewing will be used to explore individuals’ perspectives around a studied phenomenon by having an in-depth conversation [42]. Qualitative data collection will focus on three main themes: (i) the implementation of the Exemplar in Peterborough, (ii) the relationships among local service providers and (iii) the role of the patient-centred approach in the Exemplar. Semi-structured interviews are often accompanied by an interview guide to assist with focusing the discussion on selected topics [43]. Thus, an interview guide has been developed based on the three main themes of interest (see Additional file 3). Selection of interview questions was also informed by suggestions from the PPI group, professionals and the steering group coordinating the Peterborough Exemplar implementation.
  Purposive sampling will be used to select interview participants based on their relevance and richness of knowledge around the interview guide topics [44]. Staff members from service providers and funders participating in the implementation of the Peterborough Exemplar will be invited for an interview. Potential participants from four organizational levels will be invited: (i) frontline workers from primary care, social care and the third sector, (ii) frontline workers from secondary care, and (iii) managers and (iv) senior management staff from Exemplar stakeholder organizations. Each level includes professionals from different teams of the same organization or different organizations participating in the delivery of the Peterborough Exemplar. Interviewing key stakeholders across professional levels will allow the collection of rich and diverse information about the implementation process of the Exemplar.
  Focus groups could also be employed to explore professionals’ perspectives discussed in a group environment [45]. In this study, qualitative interviewing was preferred in order to reduce potential hesitancy among participants in expressing sensitive or critical views [45] in the presence of colleagues, for instance on topics about the collaboration among teams or improvements needed in the Exemplar implementation. A limitation of qualitative interviewing, however, is that topics generated from a shared understanding of participants—as can ideally happen within a focus group—may be lost [46, 47].
  Potential participants will be identified with the help of a gatekeeper from each Exemplar partner. A gatekeeper will indicate staff members who work in a service or team participating in the Exemplar (e.g. PDCS or PCMHS) or staff members whose work may be impacted by the Exemplar (e.g. GPs). Staff members whose work is not related to or impacted by the implementation of the Peterborough Exemplar will be excluded. Potential participants will receive an email invitation with a participant information sheet and a consent form developed by the lead author. One reminder will be sent to potential participants 2 weeks following the initial invitation. Participation in interviews will be anonymous and confidential. All interviews will be conducted via Microsoft Teams, and interviews will be recorded with an audio recorder. It is estimated that each interview will last between 45 and 60 minutes. Anonymized recordings will be transcribed verbatim, and recordings will be deleted following transcription completion.
  A thematic analysis will be conducted to analyse interview transcripts, a method frequently used to analyse qualitative data [48]. Thematic analysis is a method used to query a textual dataset with the aim of finding reoccurring meanings that capture topics related to the studied subject, also called “themes” [48, p. 79]. Data collection and thematic analysis will be conducted by the lead author, who has 3 years of experience on qualitative data collection and analysis, including thematic analysis. Four samples of anonymized transcripts will be discussed with GJ, KF and JP to review the coding process. GJ has received training on analysis qualitative data, and KF and JP have experience in conducting qualitative research in healthcare settings. GJ and JP’s clinical experience will also inform data analysis and interpretation. NVivo data analysis software will be used to assist with analysis of interview transcripts [49].

## Discussion

The Peterborough Exemplar components were developed based on a bottom-up approach, where service gaps and local population needs were assessed by the Exemplar developers, creating an example of a community mental healthcare model that has organically emerged from evaluating local characteristics. Given the multiple dimensions of the intervention, the evaluation required consideration of different evaluation methods and the adoption of an evaluation design that allows the evaluation of the services without experimental conditions in place. Quasi-experimental designs present several limitations with respect to internal validity, as they cannot control for all factors and other events taking place during the intervention implementation that may affect findings [50]. The most prominent event impacting the Exemplar implementation is the COVID-19 pandemic. Patient-rated questions about the impact of the pandemic are included in the analysis to evaluate the impact of the pandemic on patients’ health and service utilization. However, other factors may influence service provision—for example, other local interventions implemented during the same period in the comparison group—over which evaluators could have no control.

While it is common practice to share findings at the end of an evaluation, intermediate reporting can contribute to understanding the impact of a complex intervention and allow the consideration of interim findings in the implementation process [51]. Intermediate and final evaluation outcomes will be disseminated to local commissioners and the Exemplar partners. Evaluation outcomes will also be shared with NHS England to inform community mental healthcare and healthcare integration policies in England. Final evaluation outcomes will be published in academic journals. Final outcomes could be used to advise the implementation and evaluation of community mental healthcare models at a national and international level. However, the Exemplar structure and local context should be taken into account when considering the usefulness of the evaluation outcomes for other healthcare settings. A detailed description of the intervention group (Peterborough) and comparison group (Fenland) and the analysis conducted will be included when publishing outcomes to inform readers.

## Supplementary Information

**Additional file 1: Table S1.** Key components of the Peterborough Exemplar. A table describing the structure of the Peterborough Exemplar.

**Additional file 2.** Evaluation design of the Peterborough Exemplar. A diagram describing the methods employed for the evaluation of the Peterborough Exemplar.

**Additional file 3.** Interview guide: evaluation of the Peterborough Exemplar. The interview guide used to conduct qualitative interviews with staff members within the Peterborough Exemplar.

## Data Availability

Not applicable.

## Abbreviations

ACTs: Assertive community treatment teams
CCG: Clinical commissioning group
CPFT: Cambridgeshire and Peterborough NHS Foundation Trust
CMHTs: Community mental health teams
CRTs: Crisis resolution teams
ED: Eating disorders
EI: Early implementer
EIP: Early intervention in psychosis
GPs: General practices
IAPT: Improving Access to Psychological Therapies
LSOAs: Lower-layer super output areas
NHS: National Health Service
NIHR: National Institute for Health Research
PCMHS: Primary care mental health service
PCNs: Primary care networks
PD: Personality disorders
PDCS: Personality disorder community service
PPI: Patient and public involvement
PSS: Psychological skills services
SASPD: Standardized Assessment of Severity of Personality Disorder
STP: Sustainability and transformation plan
