# Supplementary material for: The Peterborough Exemplar: a protocol to evaluate the impact and implementation of a new patient-centred, system-wide community mental healthcare model in England
Source: Health Res Policy Syst. 2022 Feb 5;20:16. doi: 10.1186/s12961-022-00819-0 (PMC8817469; doi:10.1186/s12961-022-00819-0)
Supplement: Supplementary file 1 — Additional file 1: Table S1. Key components of the Peterborough Exemplar. A table describing the structure of the Peterborough Exemplar. [file 12961_2022_819_MOESM1_ESM.docx]

Table S1: Key components of the Peterborough Exemplar

| *Exemplar pillar* | *Organisation* | *Component* | *Area of investment* | *Description* |
| --- | --- | --- | --- | --- |
| Knowledge exchange pathways to strengthen inter-organisational relationships | Mental Health Trust  Primary Care Networks (PCN) ¹ | Virtual Clinics | A process of knowledge exchange focusing on best practice and patient management, aiming to improve clinical communication between primary health care and secondary mental health care | Virtual Clinics are online clinical meetings set up at intervals agreed between GP practices and the PCMHS. Six Primary Care Mental Health Service (PCMHS) Teams are linked with local PCNs. PCMHS operates within the Mental Health Trust with the aim of assessing referrals of patients from GPs and with dedicated time to liaise with the PCNs. Staff from the Mental Health Trust attending the clinics can also include senior nurses, consultants, a Personality Disorders specialist and a pharmacist. |
|  | Primary Care Networks (PCN) | PCN Mental Health Leads | A Mental Health Lead based in each PCN to enable collaboration between primary health care and secondary mental health care | A GP is appointed as Mental Health Lead in each PCN to liaise with a senior representative of the PCMHS with the aim of overseeing and optimise Virtual Clinics, referrals from GPs to mental health care and training where needed. Mental Health Leads are based within each PCN. |
|  | Mental Health Trust | Website | A website enabling access to information about local wellbeing and mental health support | The website brings together information about local activities, courses and services for mental health and wellbeing available to residents of the Peterborough area. The website also functions as a source for GPs, social prescribers and the Community Engagement Team. |
| Lower-threshold community services to address gaps in local service demand | Mental Health Trust | Psychological Skills Services | New psychological services to provide individual interventions and group therapy for service users with complex needs | The service includes brief psychological interventions, group therapy and recovery therapy for patients with depression, anxiety, complex trauma, and recovery needs. Services are for service users who need psychological support, but do not meet the threshold for secondary care services. |
|  | Mental Health Trust | Personality Disorders Community Services | New services for personality disorders that address a wider range of needs | The service is offering three types of group therapy to service users with personality disorders. It is an expansion of existing personality disorder services, but with a lower access threshold. |
|  | Third Sector Organisation (1) ² | Peer Support Groups for Personality Disorders | Peer Support Groups delivered by a third sector organisation run alongside the Personality Disorders Community Services | The Peer Support Groups are available for service users who join the personality disorders group therapy delivered by the Mental health Trust. The group is run parallel to the group therapy sessions and service users who access group therapy are also offered access to the Peer Support Group. |
|  | Third Sector Organisation (1) | Peer Support Groups for Rural Areas | Peer Support Groups accessible to service users in rural areas | Peer Support Groups are available for individuals with personality disorders living in rural areas of Peterborough. |
|  | Mental Health Trust  Third Sector Organisation (2) | Dual Diagnosis and Outreach Team | A dual diagnosis and outreach team supporting mental health needs of people with a dual diagnosis, including homeless people | The team works collaboratively with a third sector organisation and the local authority to offer specialist mental health support for individuals with SMIs ³. The team offers outreach treatment to people living on the streets of Peterborough with mental health problems who have not accessed mental health care services before. |
|  | Mental Health Trust | Mental Health Pharmacist | A pharmacist role advising patients, primary and secondary care professionals | A pharmacist is based in the Mental Health Trust and provides face-to-face patient support and advice, and pharmacological advice to mental health professionals, GPs, and primary care pharmacists (e.g., on prescribing and de-prescribing). The pharmacist is supported by a Strategic Pharmacist located in the local CCG who focuses on enhancing the role of pharmacies in mental healthcare across the county. |
|  | Mental Health Trust  Third Sector Organisation (3) | Community Engagement Team | A community-based team proving advice to GPs and support to service users around social prescribing | The Community Engagement Team provides advice to GPs about local community-based social prescribing interventions and assists service users with accessing them. The Team operates at a primary care level. |
|  | Mental Health Trust  Social Care | Social Care Pathway | A social care pathway operating at a primary level to support service users with SMIs whose needs are not currently pharmacological or psychological. | The social care pathway contributes to bridging the gaps between secondary mental health care and social care and supports patients with SMIs requiring social care or recovery support. The pathway offers early intervention to service users with SMIs who have social difficulties and social needs that may trigger a crisis. |

¹ Primary Care Networks (PCNs): Coalitions of General Practices (GPs) operating within a geographic area offering care tailored to the needs of local populations.

² Third Sector Organisation (1): Numbers were added next to ‘Third Sector Organisation’ to indicate different organisations.

³ SMIs: Severe Mental Illnesses
