# Supplementary material for: The Peterborough Exemplar: a protocol to evaluate the impact and implementation of a new patient-centred, system-wide community mental healthcare model in England
Source: Health Res Policy Syst. 2022 Feb 5;20:16. doi: 10.1186/s12961-022-00819-0 (PMC8817469; doi:10.1186/s12961-022-00819-0)
Supplement: Supplementary file 2 — Additional file 2. Evaluation design of the Peterborough Exemplar. A diagram describing the methods employed for the evaluation of the Peterborough Exemplar. [file 12961_2022_819_MOESM2_ESM.pptx]

## Slide 1
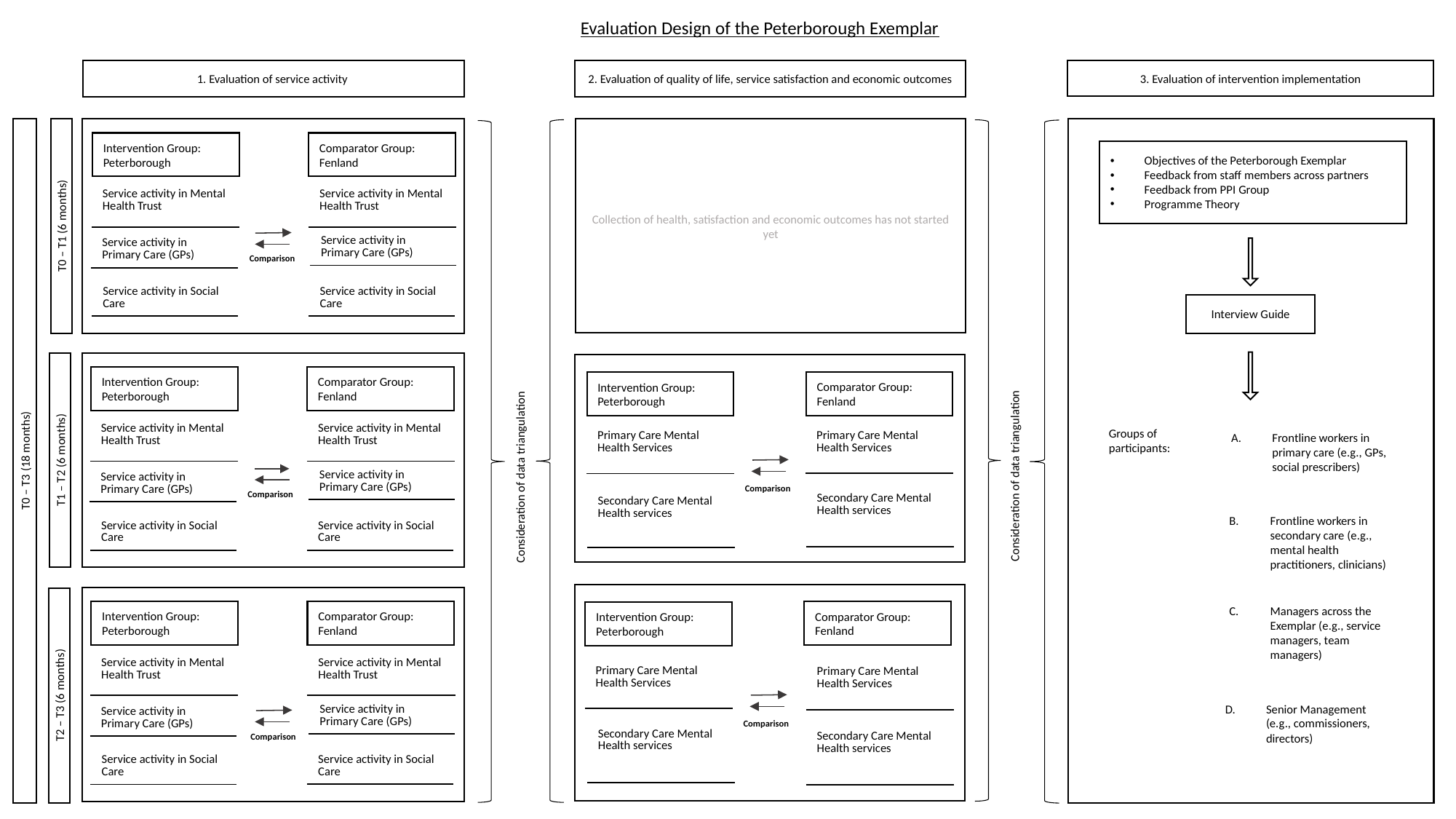

Evaluation Design of the Peterborough Exemplar
2. Evaluation of quality of life, service satisfaction and economic outcomes
1. Evaluation of service activity
3. Evaluation of intervention implementation
T0 – T3 (18 months)
T0 – T1 (6 months)
Collection of health, satisfaction and economic outcomes has not started yet
Intervention Group: Peterborough
Comparator Group: Fenland
Objectives of the Peterborough Exemplar
Feedback from staff members across partners
Feedback from PPI Group
Programme Theory
| Service activity in Mental Health Trust |
| --- |
| Service activity in Mental Health Trust |
| --- |
| Service activity in Primary Care (GPs) |
| --- |
| Service activity in Primary Care (GPs) |
| --- |
Comparison
| Service activity in Social Care |
| --- |
| Service activity in Social Care |
| --- |
Interview Guide
T1 – T2 (6 months)
Consideration of data triangulation
Consideration of data triangulation
Intervention Group: Peterborough
Comparator Group: Fenland
Comparator Group: Fenland
Intervention Group: Peterborough
Frontline workers in primary care (e.g., GPs, social prescribers)
Groups of participants:
| Service activity in Mental Health Trust |
| --- |
| Service activity in Mental Health Trust |
| --- |
| Primary Care Mental Health Services |
| --- |
| Primary Care Mental Health Services |
| --- |
| Service activity in Primary Care (GPs) |
| --- |
| Service activity in Primary Care (GPs) |
| --- |
Comparison
Comparison
| Secondary Care Mental Health services |
| --- |
| Secondary Care Mental Health services |
| --- |
Frontline workers in secondary care (e.g., mental health practitioners, clinicians)
| Service activity in Social Care |
| --- |
| Service activity in Social Care |
| --- |
T2 – T3 (6 months)
Managers across the Exemplar (e.g., service managers, team managers)
Intervention Group: Peterborough
Comparator Group: Fenland
Comparator Group: Fenland
Intervention Group: Peterborough
| Service activity in Mental Health Trust |
| --- |
| Service activity in Mental Health Trust |
| --- |
| Primary Care Mental Health Services |
| --- |
| Primary Care Mental Health Services |
| --- |
Senior Management (e.g., commissioners, directors)
| Service activity in Primary Care (GPs) |
| --- |
| Service activity in Primary Care (GPs) |
| --- |
Comparison
| Secondary Care Mental Health services |
| --- |
| Secondary Care Mental Health services |
| --- |
Comparison
| Service activity in Social Care |
| --- |
| Service activity in Social Care |
| --- |
