# Supplementary material for: The Peterborough Exemplar: a protocol to evaluate the impact and implementation of a new patient-centred, system-wide community mental healthcare model in England
Source: Health Res Policy Syst. 2022 Feb 5;20:16. doi: 10.1186/s12961-022-00819-0 (PMC8817469; doi:10.1186/s12961-022-00819-0)
Supplement: Supplementary file 3 — Additional file 3. Interview guide: evaluation of the Peterborough Exemplar. The interview guide used to conduct qualitative interviews with staff members within the Peterborough Exemplar. [file 12961_2022_819_MOESM3_ESM.docx]

# Interview Guide – Evaluation of the Peterborough Exemplar

| 1. Could you please tell me a few details about your job? 2. How does your service contribute to meeting mental health needs in Peterborough? 3. Several services have been developed within the Peterborough Exemplar. I would like to ask you about whether you work with these services and, if yes, in which way.   *Interviewer mentions key Exemplar teams, including Primary Care Mental Health Service (PCMHS), Psychological Skills Services (PSS), Personality Disorders Community Service (PDCS), ‘How Are You’ platform, secondary specialist team.* |
| --- |
| 1. How are service users involved in decisions about their treatment? 2. How are service users involved in decisions about their medication? 3. How are service users supported in their recovery (i.e., when leaving secondary care)? 4. How is information shared with the GP / mental health professional about patients’ care? 5. How do you support service users accessing mental health services? |
| 1. How do you collaborate with primary care (GPs) / secondary care to support patients? 2. How do you collaborate with social care to support patients? 3. How do you collaborate with the third sector to support patients? |
| 1. Thinking broadly about Peterborough, what kind of mental health care do you think is needed? 2. What do you think has worked well so far regarding the implementation of the Exemplar? 3. And what do you think has not worked as well so far? 4. Are there staff members that you think had a key role on the implementation of the Exemplar? If yes, can you tell me what their contribution has been? 5. How do you think COVID-19 has impacted the implementation of the PE Exemplar? 6. Do you have any recommendations about the implementation of the Exemplar? |

*Note: Interviewer may use exploratory questions where useful, e.g., ‘Can you tell me a few more details about that?’, ‘How would you explain that?’, ‘Could you please give an example?’.*
